# Special-form radial collateral artery perforator flaps for the reconstruction of complex hand defects

**DOI:** 10.1186/s13018-024-05024-z

**Published:** 2024-09-02

**Authors:** Yu Xiong, Qin Xiong, Li-Ming Qing, Pan-Feng Wu, Ju-Yu Tang, Fang Yu

**Affiliations:** Department of Hand and Microsurgery, Xiangya Hospital, Central South University, No. 87 Xiangya Road, Changsha, 410000 China

**Keywords:** Radial collateral artery perforator flap, Polyfoliate flap, Chimeric flap, Hand, Reconstruction

## Abstract

**Background:**

The reconstruction of complex wounds of the hand still has challenges in achieving aesthetic, functional and sensory recovery. We presented our experience of using the polyfoliate and chimeric radial collateral artery perforator flaps (RCAPF) to repair complex hand defects, aiming to explore the feasibility of special-form RCAPFs in hand coverage and enhance the comprehension of their respective indications.

**Methods:**

From June 2014 to March 2021, 26 cases (19 males and 7 females, mean 44.4 years) underwent defect and sensation reconstruction of their hands with special-form RCAPFs, which manifested as multiple adjacent or irregular single wounds and composite tissue defects complicated with a degree of nerve injury. The clinical effects of the free RCAPFs were evaluated by integrating the postoperative and long-term follow-up outcomes of all cases.

**Results:**

Altogether 8 polyfoliate flaps, 17 chimeric flaps and 1 polyfoliate-chimeric flap were harvested. Of them, 23 flaps survived uneventfully in one stage. Venous congestion occurred in 3 cases, two of which survived through vascular exploration and another one was finally repaired by the contralateral RCAPF. The follow-up results showed that the appearance of both the recipient and donor sites mostly recovered satisfactory. All the bone flaps properly healed. The BMRC sensory evaluation results of all skin flaps were S4 in 8 flaps, S3 in 18 flaps, and S2 in 9 flaps.

**Conclusions:**

The free RCAPFs can be designed in various forms with a reliable blood supply, contributing to reconstructing simple and multiple wounds of the hand with or without bone defects and dead space.

## Introduction

The lateral arm flap (LAF) is primarily used to cover small to moderate defects in the upper extremity as well as the head and neck with a reliable vascular pedicle [1–3]. Besides, not only can it reconstruct composite soft tissue and bone defects by carrying a humeral segment or triceps tendon, but also it can achieve sensory recovery [4, 5]. With its reliability, versatility as well as similar color and texture to the hand, the free LAF is considered as an alternative to other free flaps in repairing the wounds of the hand [6]. However, traditional LAF is usually bulky and its width for harvesting is limited [7, 8]. It’s also challenging to harvest its attached flaps when ensuring the normal blood supply and reducing the waste of the donor tissue [9, 10].

Compared with the traditional LAF, the radial collateral artery perforator flap (RCAPF) preserves the nourishing perforators and carries neither the deep fascia nor the muscle, which makes it less bulky and also alleviates the damage to the donor site [11, 12]. With the popularization of perforator flaps, they have been further improved into several special forms to achieve more individual, three-dimensional and minimally invasive wound reconstruction [13–15]. Accordingly, the polyfoliate flap corresponds to more than two of the same type perforator flaps harvested in one perforasome and is often used for simultaneous repair of two or more adjacent wounds [16, 17]. The chimeric flap consists of multiple independent tissue flaps such as skin flap, muscle flap, bone flap and fascial flap with a common pedicle, which can be used to reconstruct the wounds combined with dead space or other tissue defects [18, 19].

Given the characteristics of the RCAPFs, we designed several special-form flaps to repair some complex hand defects that were difficult for one-stage reconstruction. Our study was conducted to explore the feasibility of the special-form RCAPFs in hand coverage and enhance the comprehension of their respective indications.

## Methods

### Patients

From June 2014 to March 2021, 26 patients (19 males and 7 females) aged from 23 to 63 years (mean 44.4 years) underwent defect and sensation reconstruction of their hands with polyfoliate and chimeric RCAPFs, manifesting as multiple adjacent or irregular single wounds and composite tissue defects all complicated with the exposure of deep tissue and a degree of nerve injury.

There were 10 cases of wounds located in the left hand and 16 in the right hand, including 21 in the digits and the first web, 4 in the dorsum and 1 in the palm of the hand. Of all patients, 15 cases had both skin and phalanx defects of the thumb. Nearly a half of wounds were caused by crushing injury, and the others resulted from post-traumatic infection, traffic accident, high-pressure injection, electrical injury, etc. (Details in Table 1).

**Table 1:** Detailed data of the patients

Case	Gender /Age(Y)	Causes	Site of Defects	Size of Skin Flaps(cm)	Size of Bone Flaps(cm)	Recipient Vessels	Complications	Follow-up (M)	Hand Function	Sensory Recovery
1	M/50	Traffic Accident	Hand Dorsum-R	5 × 4; 6 × 3	—	RA`/CV	None	7	Excellent	S4; S4
2	M/27	Crush Injury	Thumb and First Web-R	8 × 4; 8.5 × 4	—	RA/CV	None	10	Good	S3; S3
3	M/55	High Pressure Injection	Hand Dorsum-L	8.5 × 5; 11 × 4.5	—	RA`/CV	None	36	Good	S3; S3
4	M/37	High Pressure Injection	Hand Dorsum-R	9 × 4; 9.5 × 4	—	RA`/CV	None	17	Excellent	S4; S3
5	M/28	Crush Injury	Middle and Ring Fingers-R	5.5 × 4; 3.5 × 6	—	CDA-II/DDV	None	18	Excellent	S3; S3
6	M/50	Hot Crush Injury	Index, Middle and Ring Fingers-L	6 × 5; 7 × 4	—	CDA-II/DDV	None	13	Excellent	S3; S3
7	M/30	Electrical Injury	Forearm and Palm-L	9 × 4; 10 × 3.5	—	RA/RV	None	7	Poor	S2; S2
8	F/30	Burn Injury	Index and Middle Fingers-L	4 × 6; 4 × 8	—	RA/RV+DMV	VC	5	Good	S2; S2
9	M/45	Post-traumatic Osteomyelitis	Thumb-L	5 × 2.5	2.5 × 1	RA`/CV	None	76	Good	S3
10	M/47	Post-traumatic Osteomyelitis	Thumb-L	4 × 2	1.5 × 0.6	RA/RV	None	55	Excellent	S4
11	M/37	Crush Injury	Thumb-R	7 × 4	2 × 1	RA`/CV	None	4	Excellent	S4
12	M/50	Crush Injury	Thumb-R	9 × 4	2 × 1	PPDA/SV	None	8	Excellent	S3
13	M/51	Chainsaw Injury	Thumb-R	10 × 4	2.5 × 1	RA`/CV	None	25	Excellent	S4
14	M/63	Avulsion	Thumb-L	18 × 5	2 × 1	RA`/CV	None	11	Excellent	S2
15	M/23	Traffic Accident	Hand Dorsum-R	11 × 5	5 × 2*	RA`/CV	None	37	Excellent	S4
16	M/39	Crush Injury	Thumb-L	9.5 × 4	2 × 0.6	RA`/CV	None	29	Excellent	S4
17	F/46	Crush Injury	Thumb-L	10 × 5	3 × 1	RA`/CV	None	17	Excellent	S3
18	F/52	Post-traumatic Osteomyelitis	Thumb-R	6.5 × 2.5	2 × 1	RA`/CV	None	15	Excellent	S3
19	M/52	Crush Injury	Thumb-R	6 × 5	2 × 1	RA/RV	None	14	Excellent	S3
20	F/50	Crush Injury	Thumb-R	6 × 3.5	2.5 × 1	RA/RV	VC	14	Good	S2
21	F/54	Cut Injury	Thumb-R	8 × 4	1.5 × 1	PPA/DDV	None	7	Excellent	S3
22	M/47	Electrical Injury	Thumb and First Web-R	8 × 5; 8 × 6.5	3.5 × 1	RA/RV	VC	8	Good	S2; S2
23	F/50	Crush Injury	Thumb-R	12 × 5	4 × 1	RA/RV	None	10	Good	S2
24	F/34	Post-traumatic Infection	Middle Finger-R	5 × 2	3 × 1	CDA-II/DDV	None	9	Excellent	S3
25	M/51	Traffic Accident	Index Finger-R	12 × 6	2 × 1*	RA/RV	None	5	Excellent	S3
26	M/57	Crush Injury	Thumb-L	7 × 4	1.5 × 1	RA`/CV	None	5	Excellent	S3

M, male; F, female; R, right hand; L, left hand; RA, radial artery; RA`, radial artery at the snuff box; CV, cephalic vein; CDA-II, the second common digital artery; DDV, dorsal digital vein; RV, radial vein; DMV, dorsal metacarpal vein; PPDA, proper palmar digital artery; SV, superficial vein; PPA, princeps pollicis artery; *, muscle flap; VC, venous congestion

All the operations were performed by the same team. This study followed the Ethics Committee guidelines of Xiangya Hospital, and all the patients have signed the informed consent.

### Operative technique

**Fig. 1:**
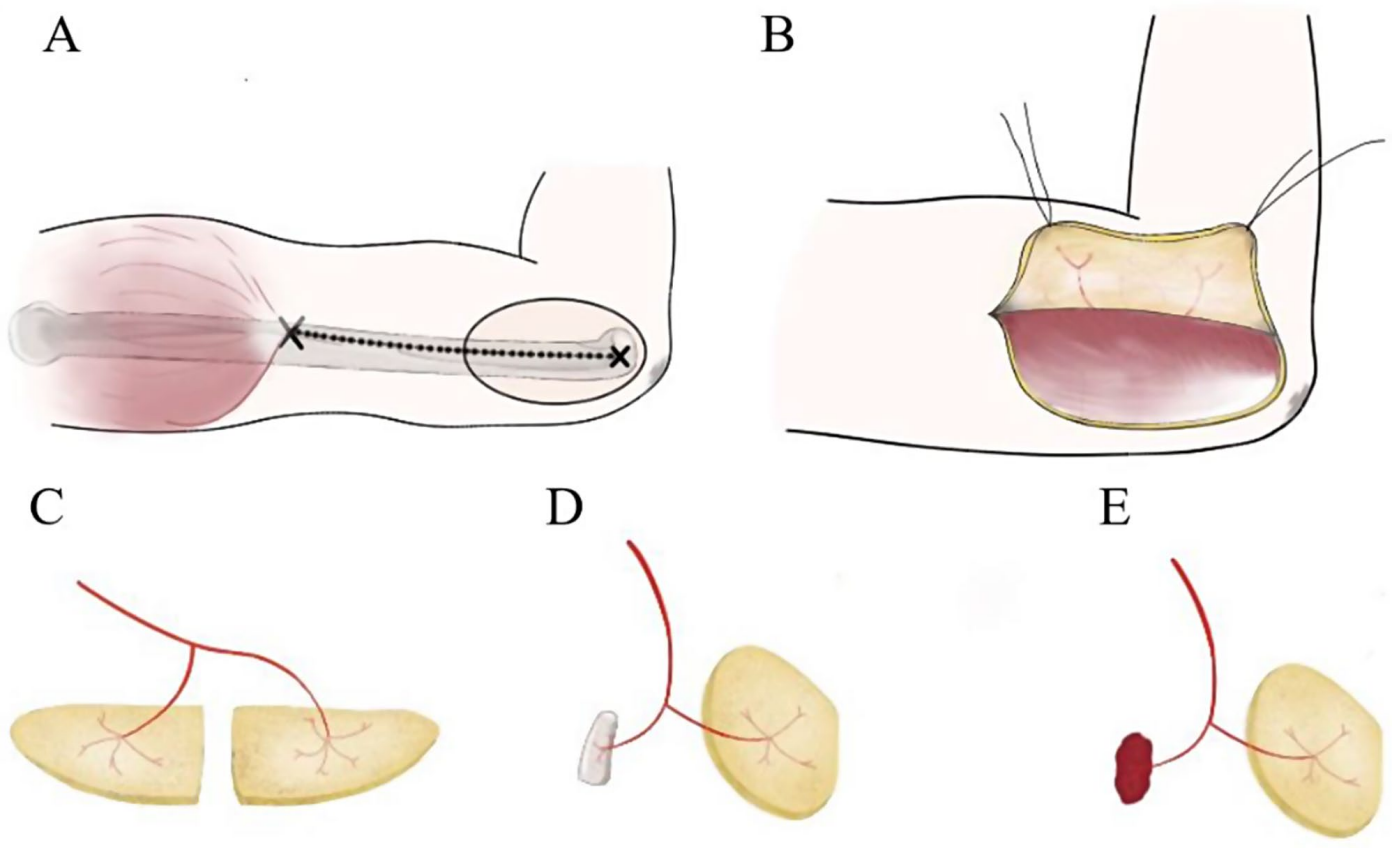
Diagram of the polyfoliate and chimeric radial collateral artery perforator flaps (RCAPF) **(A)** the axis of flap; **(B)** identified septocutaneous perforators; **(C)** polyfoliate RCAPF (double skin paddles); **(D)** chimeric RCAPF (humeral segment); **(E)** chimeric RCAPF (triceps)

After thoroughly removing the necrotic tissue, a paper template was prepared in accordance with the size and shape of the wound. It was cut and then spliced to guide the design of the skin paddles. The size of bone defect and the area of dead space were also measured for the design of an appropriate-sized bone flap or muscle flap. To ensure the direct closure of the donor site defect, a skin pinch test could be performed to evaluate the maximum available width of the flap. A line was drawn to connect the deltoid insertion and the lateral epicondyle of the humerus, which marked the lateral intermuscular septum (LIS) and the axis of the flap as well. The perforators were identified preoperatively by a hand-held Doppler along with this line (Fig. 1A).

An aseptic tourniquet was used for flap dissection. The first step was to incise perpendicularly through the skin and adipose tissue. In the suprafascial plane, the incision was performed in an anterior direction, until reaching the LIS and identifying the septocutaneous perforators from the posterior branch of the radial collateral artery (PBRCA; Fig. 1B). After dissociating the targeted perforators, the deep fascia was dissected, followed by the exposure of the PBRCA. In addition, the periosteal branches of the PBRCA that supplied the distal lateral humerus were divided to harvest a bone flap, while the branches to the triceps were identified to dissect a muscle flap (Fig. 1C, D, E).

After that, the anterior edge of the flap was incised, and then the flap was elevated from distal to proximal to dissociate the pedicle which must be separated from the radial nerve carefully. After confirming the reliable blood supply of the flap, the radial collateral artery (RCA) and its venae comitantes were ligated and then cut ultimately. The posterior cutaneous nerve of the forearm and the radial nerve should be protected and retained in the donor site. Comparatively, the posterior cutaneous nerve of the arm was carried in the flap for a further sensory reconstruction.

After transferring the flap into the recipient site, the RCA and its venae comitantes were respectively anastomosed with the corresponding artery and its venae comitantes or the cephalic vein (Table 1). The posterior cutaneous nerve of the arm could be anastomosed with the cutaneous nerve of the recipient site. The bone segment was fixed to the phalanx or the metacarpal in the site of bone defect by Kirschner wires or a plate, while the muscle flap was used to fill the dead space and prevent infection. The donor site was closed directly after complete hemostasis and drainage.

### Postoperative treatments

The blood supply of the flap needed to be monitored hourly during the first 3 days and every 4 h for 4 to 7 days after operation, including the color, temperature, extent of swelling and capillary refill. Besides, anticoagulation was routinely provided with subcutaneous low-molecular-weight heparin sodium (4000 IU/day) for a week. The injured hand was also generally immobilized for a week, followed by active flexion and extension of the affected digits.

Regular follow-ups at 1, 3, 6, 12, 24, 36 months after operation were performed to observe the appearance and texture of the flap as well as the wound healing conditions. The sensory recovery was evaluated by the sensory grading method formulated by the British Medical Research Council (BMRC) in the last follow-up, while the function of thumb opposition and the ranges of motion of the remaining four fingers were also checked.

## Results

The size of skin defects ranged from 3.0 cm × 1.5 cm to 10.0 cm × 9.5 cm, and the size of bone defects was from 1.5 cm × 0.6 cm to 4.0 cm × 1.0 cm. Altogether 35 skin flaps, 16 bone flaps and 2 muscle flaps were harvested, amounting to 8 polyfoliate flaps, 17 chimeric flaps and 1 polyfoliate-chimeric flap. The total area of polyfoliate RCAPFs with two skin paddles was from 38 to 92 cm^2^, and the area of the skin paddles in the chimeric RCAPFs was from 8 to 90 cm^2^.

There were 23 flaps surviving uneventfully in one stage. Venous congestion occurred in 3 of 26 cases at 24 h after operation, two of which survived completely after an emergent vascular exploration, whereas another one became necrotic due to a late exploration, therefore repaired with the contralateral RCAPF in a second operation. The vascular intimal injury secondary to the severe traumas induced the thrombosis of the anastomosis, which was responsible for the venous congestion of these 3 cases.

The follow-up duration lasted from 4 months to 6.3 years (mean 17.8 months). The appearance and texture of the flaps were mostly satisfactory. Sensory recovery achieved S4 in 8 flaps, S3 in 18 flaps, S2 in 9 flaps. Approximately 70% of patients showed an excellent hand function. All the bone flaps properly healed without nonunion and deformity. All the donor sites healed without any complications and only remained a linear scar (Table 1).

## Case reports

### Case 3

**Fig. 2:**
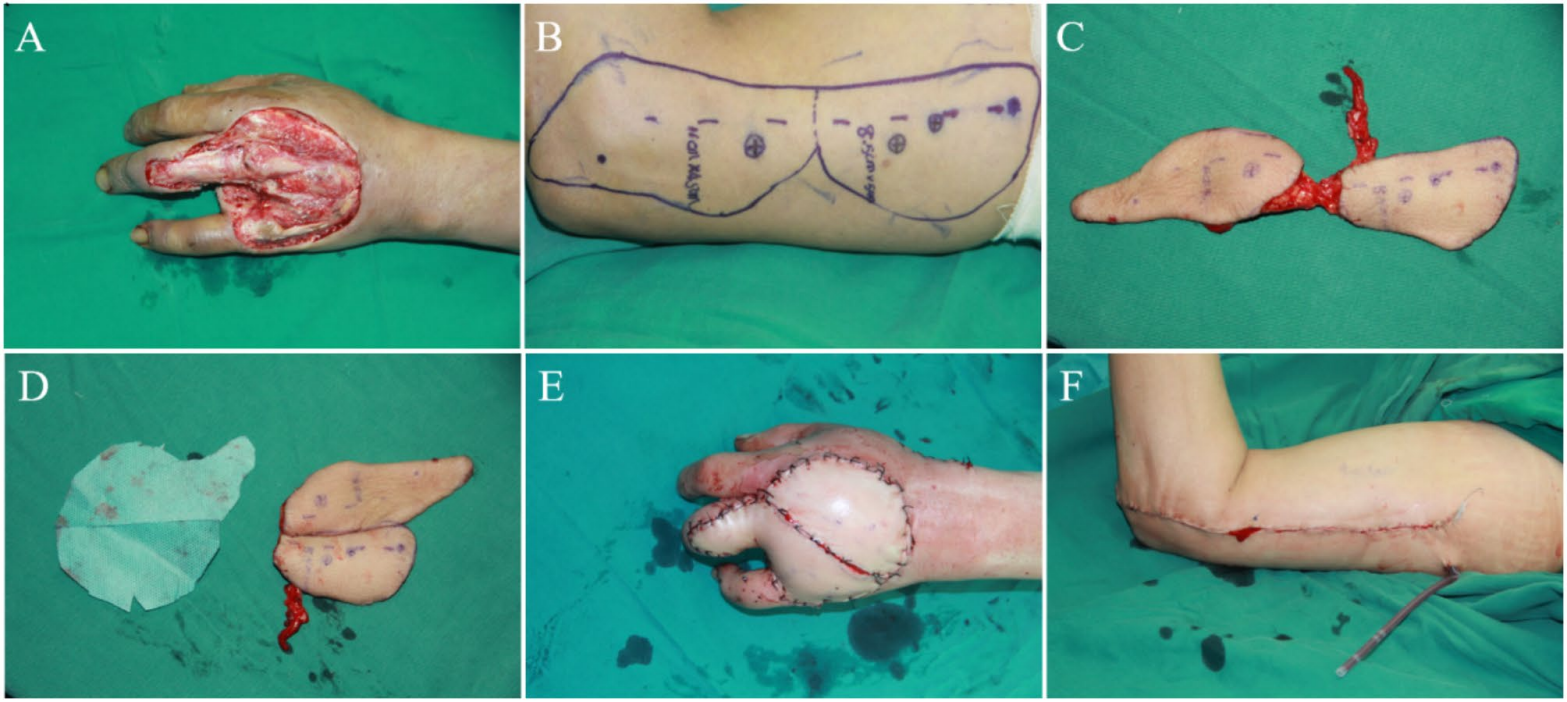
Case 3 **(A)** Wound after debridement and ring finger osteotomy; **(B)** Design of polyfoliate flap; **(C)** Complete free flaps; **(D)** Stitching flaps according to design; **(E)** Wound covered by flaps; **(F)** Direct-closed donor site

A 55-year-old male suffered a high-pressure injection injury, resulting in the soft tissue defects of the dorsum of the left hand. The extensor tendons were partially exposed, and the ring finger was completely necrotic. After a complete debridement and ring finger osteotomy, a polyfoliate RCAPF was designed for the coverage of a 9.5 cm × 10.0 cm-sized skin defect and the exposed tendons. The sizes of two skin paddles were respectively 8.5 cm × 5.0 cm and 11.0 cm × 4.5 cm. After completely harvesting the flap, the distal paddle was rotated 180 degrees and sutured with the proximal paddle side by side to cover the wound. The RCA and its venae comitantes were anastomosed with the radial vessels and the cephalic vein at the snuff box in an end-to-end manner. The donor site was closed directly. The flap survived without any complications. After 3 years of follow-up, the recipient and donor sites recovered well, and the sensory recovery of two skin paddles were both S3 (Fig. 2).

### Case 15

**Fig. 3:**
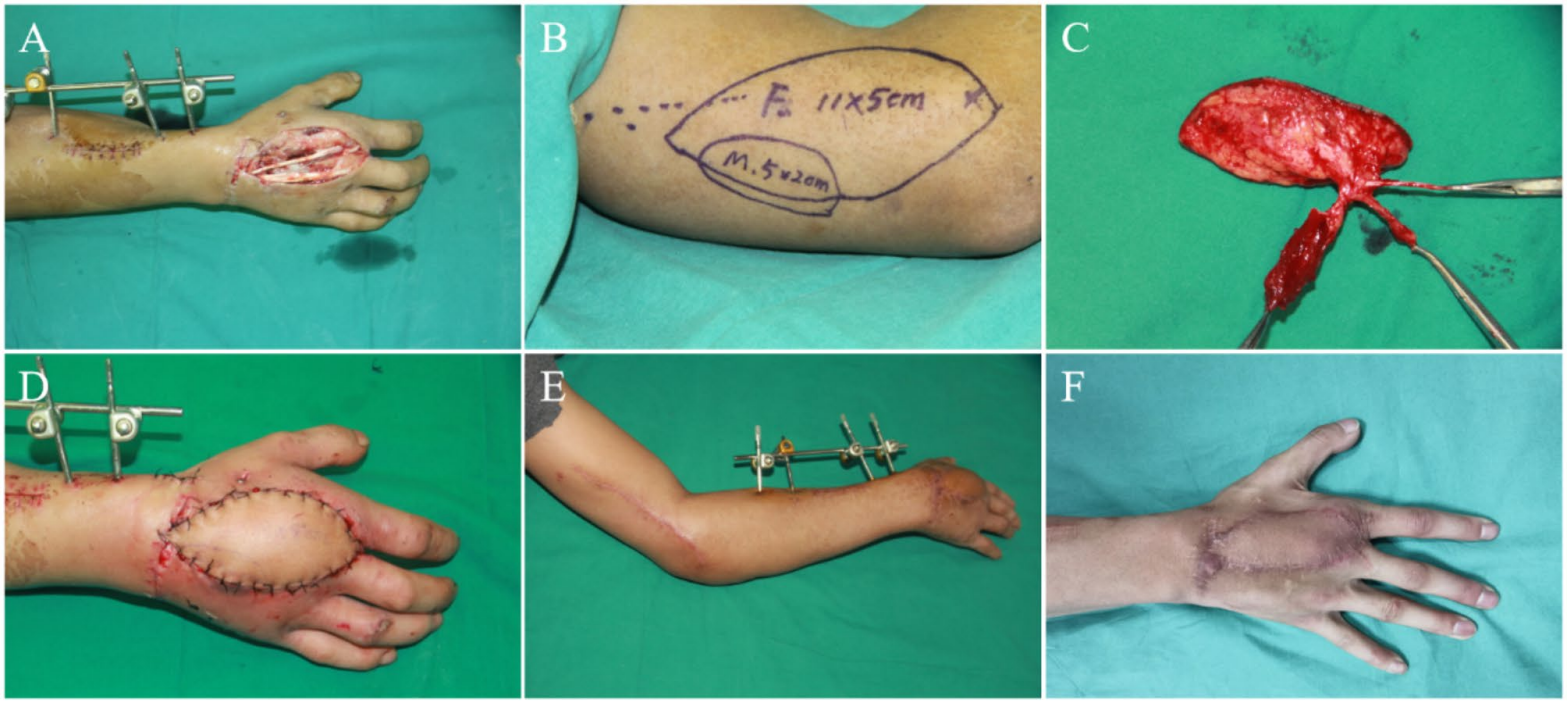
Case 15 **(A)** Wound after debridement; **(B)** Design of chimeric flap; **(C)** Complete free flaps; **(D)** Wound covered by flaps; **(E)** Appearance of donor site and recipient site at 3 months after operation; **(F)** Appearance of recipient site after 3 years of follow-up

A 23-year-old male admitted to our hospital with a 10.0 cm × 4.5 cm-sized wound of the right hand caused by a traffic accident. We designed a chimeric RCAPF with a muscle flap to cover the exposed extensor tendons and simultaneously fill the dead space. The size of the skin flap was 11.0 cm × 5.0 cm, and the size of the muscle flap was 5.0 cm × 2.0 cm. The RCA and its venae comitantes were anastomosed with the radial vessels and the cephalic vein at the snuff box in an end-to-end manner. The chimeric flap survived uneventfully without any infection. Followed up for 3 years, the appearance of the right hand was satisfactory, and the sensory recovery of the flap achieved S4. The donor site healed primarily with a linear scar (Fig. 3).

### Case 17

**Fig. 4:**
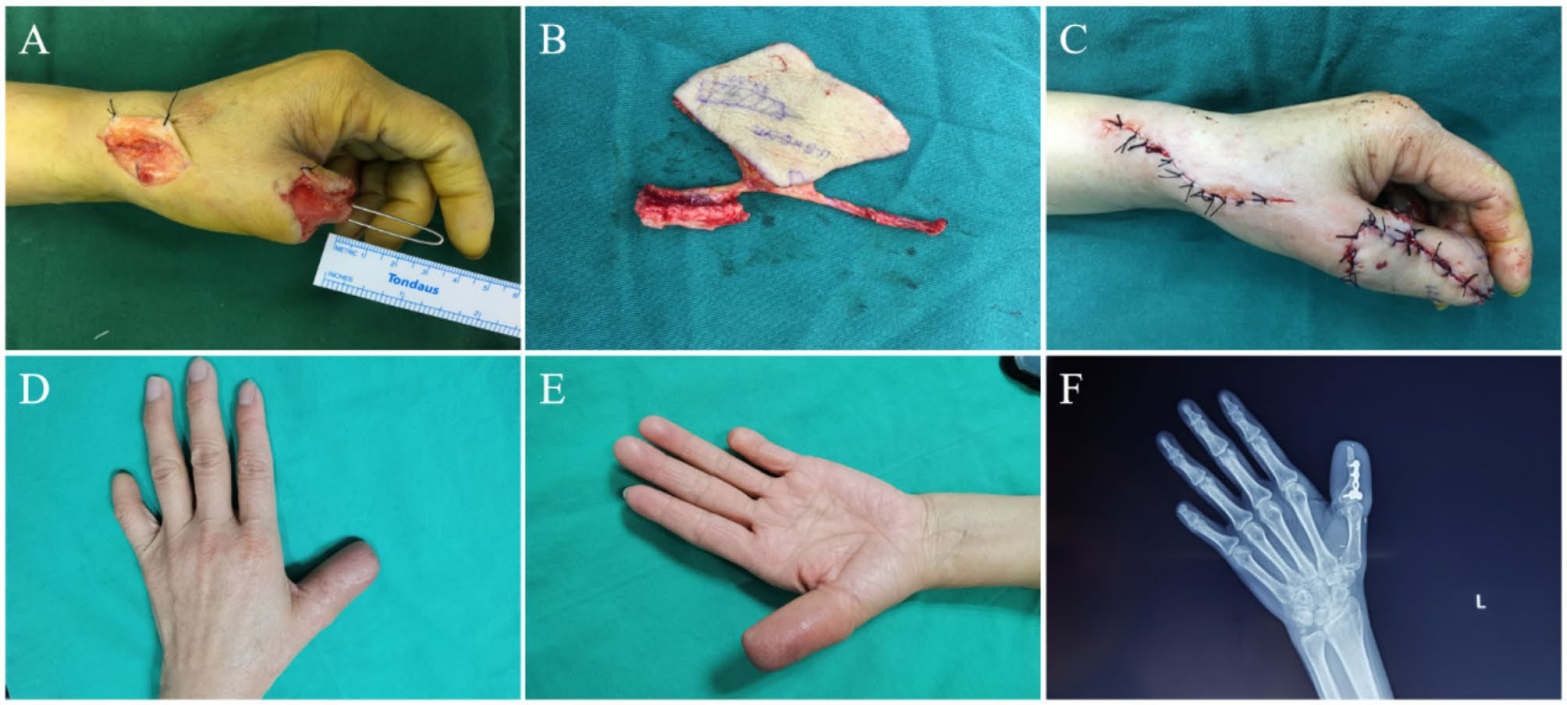
Case 17 **(A)** A 3 cm-long phalanx defect of left thumb after debridement; **(B)** Harvested chimeric flap; **(C)** Recipient site after operation; (**D**, ** E**) Dorsal view and palmar view of the recipient site after 17 months of follow-up after operation; (**F**) X-ray showed the bone flap with a plate

A 46-year-old female suffered from a crushing injury, leading to both the skin and bone defects of the left thumb. We found a 3 cm-long phalanx loss and a 9.0 cm × 4.5 cm-sized skin defect of the thumb after a thorough debridement. And then a chimeric RCAPF with a humeral flap was transferred to reconstruct the thumb. The size of the skin flap was 10.0 cm × 5.0 cm, and the size of the bone segment was 3.0 cm × 1.0 cm which was fixed to the proximal phalanx with a plate. The RCA and its venae comitantes were anastomosed with the radial vessels and the cephalic vein at the snuff box in an end-to-side manner. After 17 months of follow-up, the bone flap healed and achieved a radiologic union. The sensation of the flap returned to S3, and both the shape and function of the thumb was almost restored (Fig. 4).

## Discussion

The reconstruction of complex wounds of the hand still has challenges in achieving aesthetic, functional and sensory recovery [20]. The skin grafts, pedicled flaps and free flaps are usually used for wound coverage, some of which cannot meet such an overall requirement [6, 20]. The LAF was first presented by Song et al. [21] in 1982 as a septocutaneous flap. Subsequently, there were lots of anatomical studies and clinical reports demonstrating that it could reconstruct composite defects and sensation as a versatile flap [1, 4, 5]. Combined that with its color, texture and available amount of tissue, the free LAF is considered suitable for repairing small to moderate defects of the hand [6]. However, with the deep fascia and subcutaneous tissue involved, traditional LAF was too bulky and often needed a second-thinning procedure [7]. There were also excessive fascia and septum retained to ensure a reliable blood supply for the bone flap [3, 22].

The RCAPF, a perforator flap optimized from the LAF, is characterized by only preserving the perforators and carrying neither the deep fascia nor the muscle. The perforator propeller flap was usually rotated to cover the defects of the elbow [11, 23], whereas the free flap was mostly used to repair wounds of the hand as well as the head and neck [12, 19]. Generally, traditional perforator flaps still have limitations in repairing more complex and extensive wounds. In order to maximize the normal appearance and function of both the recipient and donor sites, they have been developed into several special forms such as polyfoliate, chimeric, microdissected, flow-through and conjoined flaps and the corresponding derivatives, contributing to the precise reconstruction of such complex defects and minimizing the damage to the donor site [13–16].

The RCA mainly derives from the profounda brachii artery and continues to divide into one posterior and one anterior branch. The PBRCA eventually anastomoses with the radial recurrent artery around the lateral epicondyle of the humerus. Crucially, the PBRCA travels through the LIS where it gives off 3 to 5 septocutaneous perforators to supply the LAF [1, 22, 24, 25]. And it has also been found that the PBRCA provides muscular branches and one group of direct periosteal branches to respectively supply the triceps and the lateral humerus [22]. Haas et al. [5] described that the constant branches to the humerus were found 3 to 6 cm proximal to the lateral epicondyle. The PBRCA simultaneously gives off reliable cutaneous, periosteal, muscular and fascial perforators [4, 9, 26], and there is a constant pedicle which can be extended more proximally for a longer length and a larger caliber [3, 12], laying the foundation for the design of special-form RCAPFs with several skin paddles or other tissue flaps.

Except for the above anatomical characteristics, the RCAPF still has many other advantages. Like the traditional LAF, the ipsilateral upper arm can be chosen as the donor site, avoiding a position adjustment during the operation. The posterior cutaneous nerve of the arm carried in the flap can provide sensory innervation to the skin of the recipient site [1, 27]. Furthermore, the RCAPF carries neither the deep fascia nor the muscle and the adipose tissue can be thinned as needed [19], which is the most distinctive difference from the traditional fasciocutaneous and myocutaneous flaps, ensuring the aesthetics of the recipient site and diminishing the donor site morbidity. The dominant vessels are not involved in the free RCAPF transfer, and the pedicle and perforators of this flap are constant and relatively easy to be dissected [3].

Through anastomosing one group of source vessel, the special-form perforator flaps with two or more tissue flaps can reconstruct complex hand defects in one-stage procedure. For the optimized polyfoliate RCAPF, it can be divided into several skin paddles to cover the multiple adjacent or multi-digital defects [17, 27]. The design of converting the flap width to the length not only helps to cover the irregular wounds more flexibly, but also allows for direct closure of the donor site defect, thereby avoiding a second-stage skin grafting or damaging the second donor site [12]. Comparatively, the chimeric flap can repair complex wounds complicated with phalanx or metacarpal defects, tendon injuries and dead space formation, realizing three-dimensional reconstruction of composite tissue defects of the hand. Each of the tissue flaps has an independent perforator with a little soft tissue cuff attached, which can increase the mobility between them [19].

It should be emphasized that the RCAPF is harvested from the outside of the upper arm and hence is not applicable to all the patients due to the individual difference of skin hairiness and thickness of the upper arm. Compared with the anterolateral thigh flap and the parascapular flap, the RCAPF is also not suitable for repairing large and extensive wounds, on account of the limited area of the donor site [6, 28]. Sensory disorders such as numbness and paresthesia may occur in the distribution area of posterior cutaneous nerves owing to the division of them [7, 28]. Sometimes the caliber of the pedicle is relatively small, which is not conductive to the microvascular anastomosis, therefore the pedicle needs be divided more proximally to the level of the profounda brachii artery [12]. Moreover, it requires higher anatomical and microanastomosis ability for the operators to perform the free RCAPF transfer and avoid the twisting of the pedicle.

What’ more, it is found that the RCAPF has a consistent blood supply from the PBRCA, and the venous drainage mainly depends on its venae comitantes. Hence, the cephalic vein is generally not necessary to be carried in the RCAPF to improve its vein drainage [29]. In Regard to the selection of recipient vessel, the branch of radial artery at the snuff box is beneficial to the anastomosis with the RCA because of its relatively small caliber. Besides, it is essential to get a sense of the latent damage of such severe traumas to the recipient vessels. And the rigorous monitoring of the blood supply of the flap and timely exploration are also vital to the prevention of vascular compromise.

The limitations in this study firstly involve the absence of postoperative evaluation standards for the hand function and objective assessment of the aesthetic outcomes. Whether the appearance and texture of the flap is satisfactory depends upon the subjective judgement of the surgeons. Secondly, there is a lack of control group and strict inclusion and exclusion criteria for this retrospective case series, which indeed requires to be taken into consideration for further case-control studies.

## Conclusions

The free RCAPFs can be designed in various forms with a reliable blood supply. In one perforasome, the polyfoliate RCAPF can be designed to repair multiple adjacent or irregular single wounds of the hand, whereas the chimeric flap can reconstruct the phalanx loss and eliminate the dead space when covering the skin defect simultaneously. The clinical applications of the special-form RCAPFs will be of great significance for the reconstruction of complex hand defects.

## Data Availability

The datasets used and/or analysed during the current study are available from the corresponding author on reasonable request.

## Abbreviations

LAF: lateral arm flap
LIS: lateral intermuscular septum
PBRCA: posterior branch of the radial collateral artery
RCA: radial collateral artery
RCAPF: radial collateral artery perforator flap
